# Dual-targeting cyclic peptides of receptor-binding domain (RBD) and main protease (Mpro) as potential drug leads for the treatment of SARS-CoV-2 infection

**DOI:** 10.3389/fphar.2022.1041331

**Published:** 2022-10-19

**Authors:** Zhen Xu, Yunting Zou, Xi Gao, Miao-Miao Niu, Jindong Li, Lu Xue, Su Jiang

**Affiliations:** ^1^ Institute of Clinical Medicine, The Affiliated Taizhou People’s Hospital of Nanjing Medical University, Taizhou, China; ^2^ Department of Pharmaceutical Analysis, China Pharmaceutical University, Nanjing, China; ^3^ Department of Pharmacy, The Affiliated Taizhou People’s Hospital of Nanjing Medical University, Taizhou, China

**Keywords:** SARS-CoV-2, receptor-binding domain, main protease, dual inhibitors, virtual screening

## Abstract

The receptor-binding domain (RBD) and the main protease (Mpro) of severe acute respiratory syndrome coronavirus 2 (SARS-CoV-2) play a crucial role in the entry and replication of viral particles, and co-targeting both of them could be an attractive approach for the treatment of SARS-CoV-2 infection by setting up a “double lock” in the viral lifecycle. However, few dual RBD/Mpro-targeting agents have been reported. Here, four novel RBD/Mpro dual-targeting peptides, termed as MRs 1-4, were discovered by an integrated virtual screening scheme combining molecular docking-based screening and molecular dynamics simulation. All of them possessed nanomolar binding affinities to both RBD and Mpro ranging from 14.4 to 39.2 nM and 22.5–40.4 nM, respectively. Further pseudovirus infection assay revealed that the four selected peptides showed >50% inhibition against SARS-CoV-2 pseudovirus at a concentration of 5 µM without significant cytotoxicity to host cells. This study leads to the identification of a class of dual RBD/Mpro-targeting agents, which may be developed as potential and effective SARS-CoV-2 therapeutics.

## Introduction

Severe acute respiratory syndrome coronavirus 2 (SARS-CoV-2) has spread rapidly across the world since its emergence in December 2019, resulting in the global pandemic of coronavirus disease 19 (COVID-19) (Chan et al., 2020; Coronaviridae Study Group of the International Committee on Taxonomy of Viruses, 2020). As of 28 August 2022, over 598 million confirmed cases and over 6.4 million deaths have been reported globally (WHO, 2022). Although COVID-19 vaccination has proven useful in reducing the risk of SARS-CoV-2 infection, severe clinical outcomes, and mortality of COVID-19, the efficacy of which is limited due to the high mutation frequency of SARS-CoV-2 (Aydogdu et al., 2022; Thakur et al., 2022). The omicron variant has recently been identified, which has led to a surge of infections and deaths due to its increased binding affinity and immune escape (Callaway, 2022; Cao et al., 2022; Tuekprakhon et al., 2022). Therefore, there is an urgent need for intervention strategies to control this public health crisis (Lino et al., 2022; Viana et al., 2022). In particular, the development of specific antiviral agents targeting the vital intervention point in the SARS-CoV-2 lifecycle is extremely attractive and promising (Sviridov et al., 2020).

The SARS-CoV-2 virion contains four structural proteins, including spike (S), membrane (M), envelope (E), and nucleocapsid (N) proteins (Chen et al., 2020). Viral entry is the first step in the SARS-CoV-2 lifecycle, which is mediated by the S protein (Peng et al., 2021; Jackson et al., 2022). The S protein of SARS-CoV-2 is a homotrimeric class I fusion glycoprotein that is made up of two essential subunits termed as S1 and S2 (Sternberg and Naujokat, 2020; Turoňová et al., 2020). Previous studies have demonstrated that the receptor-binding domain (RBD) in the S1 subunit interacting with the host-cell receptor angiotensin-converting enzyme 2 (ACE2) is critical for viral entry because it triggers the membrane fusion mediated by S2 subunit between virus and host cell (Lan et al., 2020; Zhou et al., 2020; Lee et al., 2022). In light of the important role in the first stage of SARS-CoV-2 infection, RBD is a vital target for intervention (Tai et al., 2020). Many effective approaches have been developed for blocking the interaction between RBD and ACE2, including vaccines, peptide analogues, monoclonal antibodies, protein chimeras, and small molecule inhibitors (Liu et al., 2022). However, mutations of SARS-CoV-2 mostly occurred in its RBD, which led to enhanced binding affinity to the host receptor, increasing infectivity of SARS-CoV-2 and rendering some RBD-targeted therapies invalid (Li et al., 2020; Wrapp et al., 2020). Therefore, opportunities always side with challenges in the development of anti-COVID-19 inhibitors targeting RBD.

**FIGURE 1:**
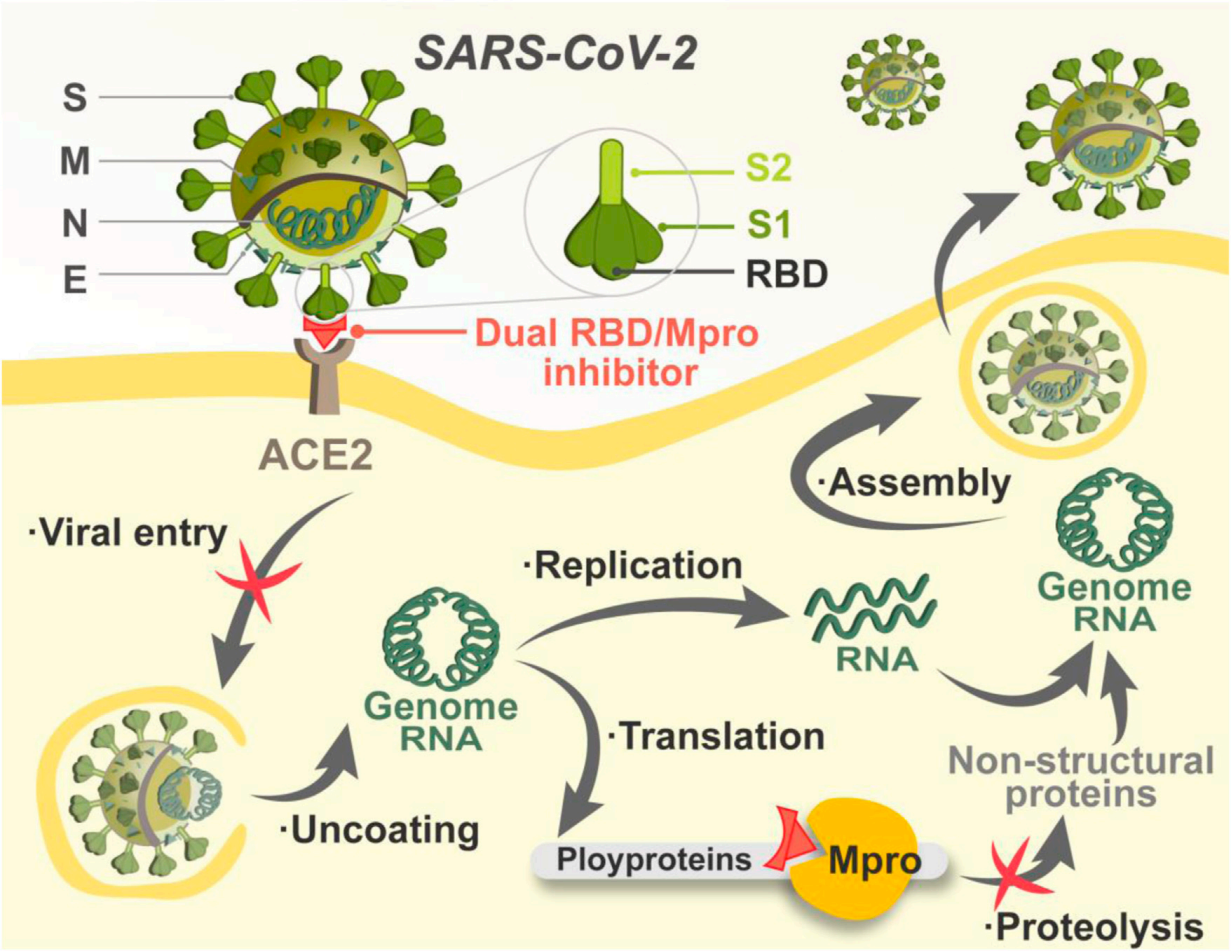
Schematic illustration of the mechanism of dual RBD/Mpro-targeting inhibitor.

Once entering host cells, SARS-CoV-2 uncoats and is ready for transcription and translation. The main protease (Mpro) is responsible for mediating the replication and transcription of SARS-CoV-2 (Dai et al., 2020). By acting on at least 11 different proteolytic sites, Mpro cleaves viral polyproteins, releasing mature proteins for viral replication (Kneller et al., 2020). Notably, the sequence of Mpro is of specificity that molecules structurally mimicking the cleavage sites can precisely target Mpro with little adverse impact on host cellular proteases (Zhang et al., 2020; Chan et al., 2021). In addition, mutations in Mpro are often lethal to SARS-CoV-2, which reduces the risk of Mpro-targeted drug resistance mediated by mutations (Goyal and Goyal, 2020). Researchers have focused on the development of Mpro inhibitors, and some advances have been made. Two peptidomimetic Mpro inhibitors discovered by Pfizer, lufotrelvir and nirmatrelvir, have entered clinical trials, and the latter recently received emergency use authorization. Both of them feature an electrophilic warhead that forms a covalent bond to SARS-CoV-2 Mpro, respectively (Owen et al., 2021; Vandyck and Deval, 2021). Nonetheless, covalently binding may cause potential off-target toxicity (Rossetti et al., 2022). Therefore, the search for Mpro inhibitors against COVID-19 is far from over, and non-covalent inhibitors are needed for the circumvention of the toxicity issue. Given that RBD and Mpro are critical for the establishment of successful entry and replication by SARS-CoV-2, non-covalent targeting of both is expected to achieve efficient inhibition against COVID-19 by setting up a “double lock” in the viral lifecycle (Figure 1).

Cyclic peptides have several favorable properties such as strong binding affinity, target selectivity, good biocompatibility and low toxicity, which make them an attractive modality for the development of therapeutics (Kreutzer et al., 2021). Docking-based virtual screening (DBVS) is a fast and efficient technique that has great potential to identify novel active peptides in the pharmaceutical industry compared with traditional high-throughput screening (Yang D. S. et al., 2020; Moreira et al., 2022). In addition, molecular dynamics (MD) simulation can greatly reflect the stability of protein-ligand interactions (Zinovjev and van der Kamp, 2020). Both methods play an important role in the screening process of drug discovery. Herein, we reported a combinatorial screening of non-covalent cyclic peptides targeting both RBD and Mpro by combining molecular docking and MD simulation. RBD/Mpro dual-targeting docking with high accuracy was used to search against a virtual peptide library. Subsequently, four hit peptides with lower docking binding energies were subjected to MD simulations for estimating the stability of protein-peptide systems. Finally, four novel peptides were confirmed to efficiently and specifically target both RBD and Mpro by *in vitro* bioassays. Furthermore, all of them effectively inhibited SARS-CoV-2 pseudovirus infection with negligible cytotoxicity to host cells and, therefore, will serve as a good starting point for structural optimization to develop dual RBD/Mpro-targeting inhibitors with improved efficacy.

## Materials and methods

### Virtual screening

The docking module in MOE (Molecular Operating Environment program) was applied to molecular docking-based screening. The X-ray 3D structures of Mpro (PDB ID:7RNW) and RBD (PDB ID:6M0J) were obtained from RCSB Protein Data Bank. Prior to docking, QuickPrep module in MOE was used to optimize the protonation state of two proteins and add hydrogens atoms. The QuaSAR-CombiGen module of the MOE program was employed to generate a fully-enumerated combinatorial library from a set of peptide fragments. Here, the QuaSAR-CombiGen enumerated a virtual library of all peptides that were combinatorially generated from three peptide fragments including cyclopeptides (containing 15 amino acids), heptapeptides and linear-peptides (containing 16 or 18 amino acids). The oxygen atom on the C-terminal hydroxyl group of each cyclopeptide and heptapeptide fragments was labeled as the “A1” port, while the N-terminal hydrogen atom of each heptapeptide and linear-peptide fragments was labeled as the “A0” port. The entire combinatorial library was enumerated by exhaustively cycling through all combinations of the peptide fragment at attachment “A1” port and other fragments at attachment “A0” port. The virtual library containing 27,000 cyclic peptides was written to an output database. Energy Minimization algorithm in MOE was utilized to convert 2D structure of each peptide to 3D structure. After that all peptides were firstly docked into the Mpro active site by the means of Dock program of MOE, the peptides with docking scores < −13.7 were further selected to dock into the active site of RBD. Based on the dG scores of peptides for RBD, the top four peptides were chosen for further study.

### MD simulations

Each protein-peptide complex was subjected to MD simulation using GROMACS 2019.4 with periodic boundary conditions to explore the stability of system evolved with time. The topology file of each complex system was built with Amber99sb-ildn force field. Each Mpro-peptide system was placed in a cubic box of size 62 Å × 50 Å × 68 Å and each RBD-peptide system was placed in a cubic box of size 41 Å × 53 Å × 63 Å. Then, each system was solvated with simple point charge (SPC) water molecules. The counter ions were added to the system to keep it neutral followed by energy minimization of complex system using steepest descent method. NVT simulation of each complex system was done at 300 K with a V-rescale thermostat for 100 ps and later NPT simulation was carried out with a Parinello–Rahman barostat for another 100 ps to maintain the pressure (1 atm). Finally, each complex system was simulated for 100 ns and trajectory data were recorded every 100 ps for further root-mean-square-deviation (RMSD) and root-mean-square fluctuation (RMSF) analysis.

### Microscale thermophoresis (MST) analysis

To detect the interaction between the peptides and RBD/Mpro, the MST experiments were performed using the Monolith NT.115 instrument. RBD and Mpro proteins were labeled with the Lys labeling kit, respectively. In the assay, the final concentration of the labeled protein was 50 nM, and the peptides were gradient-diluted in 1:1 dilution and starting from 12.5 μM. After brief incubation in MST buffer, the samples were loaded into MST-standard glass capillaries. All the measurements were performed in triplicate using automatically assigned 50% MST power and 20% LED power.

### Cell culture

Human Embryonic Kidney 293T cells (293T), human pulmonary alveolar epithelial cells (A549) and human fetal small intestinal epithelial cells (FHs 74 Int) were obtained from American Type Culture Collection (ATCC) (Manassas, VA, United States), human cardiomyocyte cells (AC16) and human brain endothelial cells (hCMEC/D3) cells were obtained from Millipore (NY, United States). All cell lines are cultured in Dulbecco’s modified Eagle’s medium (DMEM) with 1% penicillin–streptomycin and 10% fetal bovine serum (FBS) in a humidified atmosphere (95%), 5% CO_2_ at 37°C.

### Pseudovirus infection and treatment

SARS-COV-2 pseudovirus was used to evaluate the inhibitory effects of peptides on pseudovirus infection. The pseudovirus is a HIV-1 microbial particle containing a SARS-CoV-2 surface spike protein and a luciferase reporter gene so that when the pseudovirus enters a host cell, the luciferase is expressed. 293T-ACE2+ cells were seeded in 96-well plates at a density of 2 × 10^4^ cells/well at 37°C overnight. The diluted peptides were pre-incubated with the pseudovirus for 1 h and were then added to 293T-ACE2+ cells. The infected cells were washed three times and incubated for another 48 h at 37°C, and the luciferase activity was measured by using a luciferase detection kit according to the manufacturer’s instructions.

### Enzymatic assay

According to a previously reported method, (Du et al., 2021) the FRET-based enzymatic assay was used to evaluate the inhibitory effects of the peptides on Mpro. First, the Mpro (250 nM at a final concentration) was incubated with various concentrations of tested peptides in 90 μl reaction buffer for 30 min in a black 96-well plates, and then the reaction was initiated by adding 10 μl of 50 nM FRET-based peptide substrate (Dabcyl-KTSAVLQ/SGFRKME-Edans). The reaction was monitored for 1 h, and the initial velocity was calculated using the data by linear regression. The IC_50_ was calculated by plotting the initial velocity against various concentrations of testing inhibitor by using a four parameters dose−response curve in Prism software.

### Cytotoxicity assay

The suspension of cells at a concentration of 5 × 10^4^/ml were seeded into a 96-well plate. After incubation at 37°C for 24 h, the original culture medium was removed, and different concentrations of peptides were added to each well. After incubation at 37°C for 48 h, MTT stock solution (0.5 mg/ml) was added to each well and the plates incubated for 4 h. Absorbance of each well was measured at 570 nm using a microplate reader.

## Results

### Virtual screening

**FIGURE 2:**
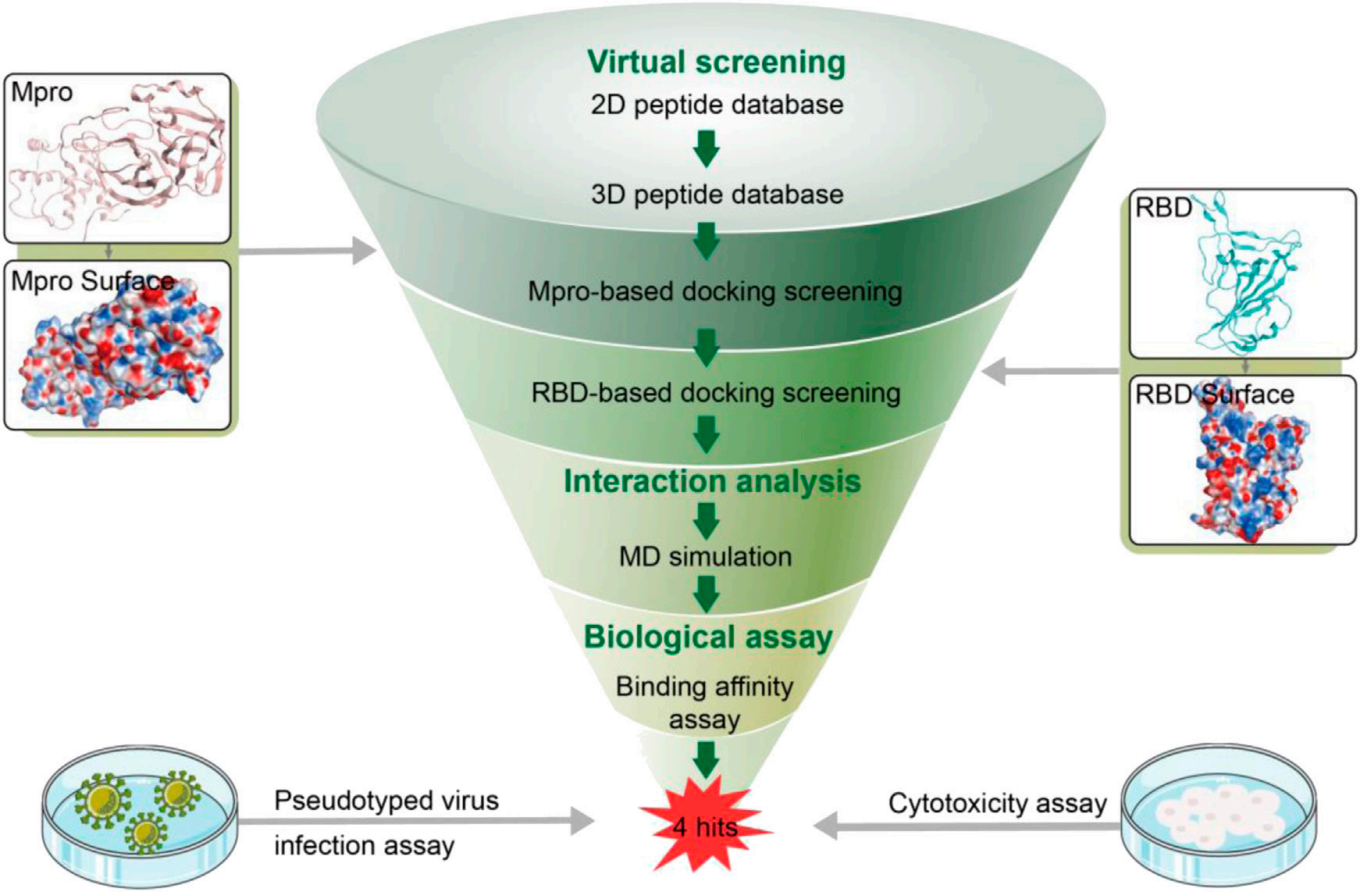
Workflow of the combinatorial screening process of dual RBD/Mpro-targeting inhibitors.

In this work, a combinatorial virtual screening protocol coupling molecular docking-based screening with MD simulation was utilized to discover potential dual RBD/Mpro inhibitors efficiently from a self-built peptide database. The process of the combinatorial screening was expressed in Figure 2. The α-helix of ACE2 has been found to be important in the interaction with RBD (Han and Král, 2020). Hence, a two-dimensional (2D) peptide database including 27,000 cyclic peptides with α-helix motif was constructed for virtual screening and the 2D structure of each cyclic peptide in database was switched to a 3D structure. Then, a molecular docking-based screening was performed against Mpro (PDB ID:7RNW) and RBD (PDB ID:6M0J). Firstly, 3D peptides were docked into the active site of Mpro to screen Mpro-targeted peptides. The docking binding energy was utilized to measure the binding affinity between the docking peptide and catalytic active site pocket of target (the more negative values indicating the better binding affinity). A reasonable docking binding energy threshold of <−13.7 kcal/mol was applied to the Mpro-targeted peptides selection, engendering the identification of 164 peptides. Subsequently, these screened peptides were docked into the active site of the RBD to identify dual RBD/Mpro peptides. The criteria to assess the binding affinity of peptide for RBD was the docking binding energy mentioned above. Finally, the top four candidate peptides (termed as MRs 1–4) with low RBD binding energies (<−13.7 kcal/mol) were selected for further study (Supplementary Table S1).

### Interaction analysis

**FIGURE 3:**
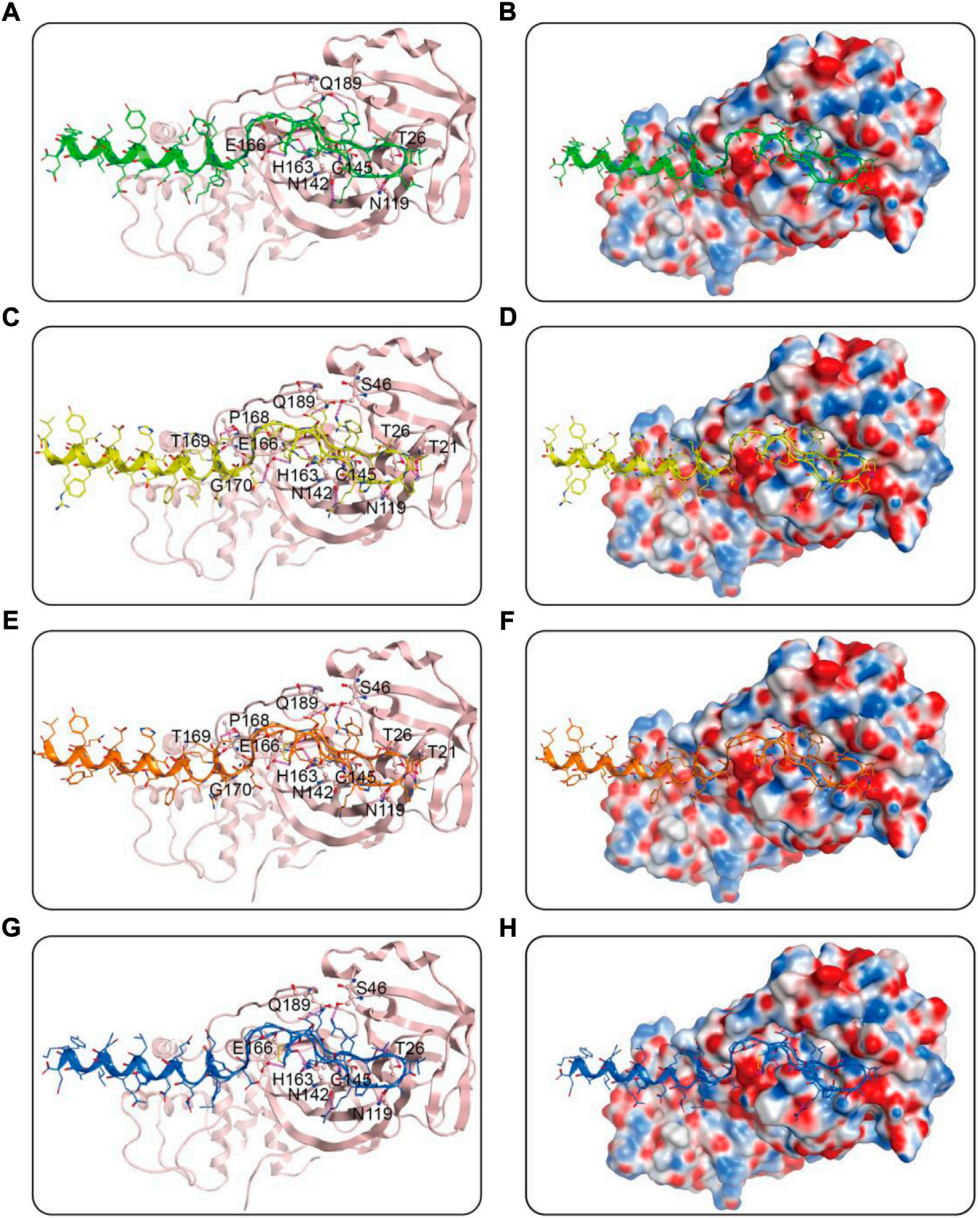
Predicted binding modes of MRs 1-4 in the active site of Mpro: **(A**,**B)** MR-1; **(C**,**D)** MR-2; **(E**,**F)** MR-3; **(G**,**H)** MR-4. The MRs 1-4 were represented as sticks with the atoms colored as nitrogen-blue, oxygen-red, sulfur-yellow and carbon-green, yellow, orange, and dark blue, respectively. Residues close to the MRs 1-4 were depicted as sticks with the atoms colored as carbon-light pink, nitrogen-blue, and oxygen-red. The hydrogen-bond interactions with protein residues were represented by purple dotted lines.

**FIGURE 4:**
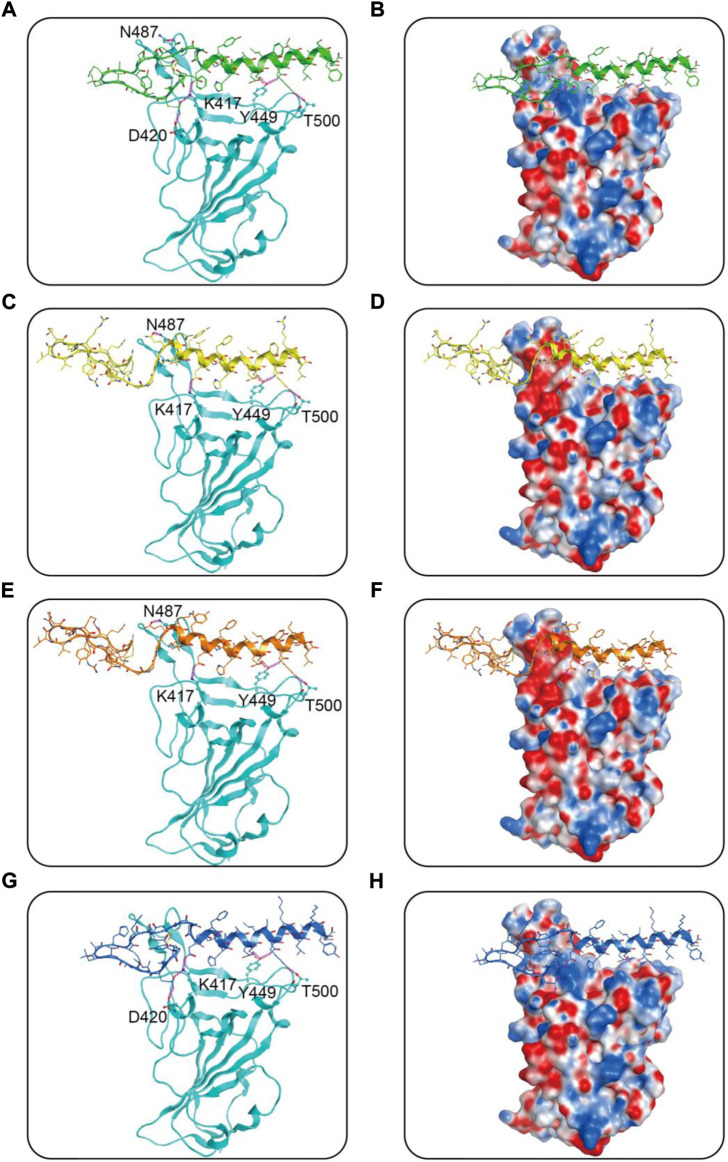
Predicted binding modes of MRs 1-4 in the active site of RBD: **(A**,**B)** MR-1; **(C**,**D)** MR-2; **(E**,**F)** MR-3; **(G**,**H)** MR-4. The MRs 1-4 were represented as sticks with the atoms colored as nitrogen-blue, oxygen-red, sulfur-yellow and carbon-green, yellow, orange, and dark blue, respectively. Residues close to the MRs 1-4 were depicted as sticks with the atoms colored as carbon-azure, nitrogen-blue, and oxygen-red. The hydrogen-bond interactions with protein residues were represented by purple dotted lines.

We then performed docking studies to investigate the binding modes of MRs 1-4 to both Mpro and RBD proteins. Figure 3 displayed the non-bonded interactions between MRs 1-4 and Mpro. As shown in Figures 3A,C,E,G, the cyclic region of each peptide makes a major contribution to binding to Mpro. All of the four peptides formed conventional hydrogen bonds with seven residues (T26, N119, N142, Cys145, E166, Q189, H163). These hydrogen bond interactions could stabilize the peptides at the active site of Mpro and thereby assist in enhancing the biological activity of the protein-peptide complexes. Notably, Cys145 has been reported as a key residue that mainly participated in hydrogen bond interaction to prevent the virus from cleaving polyprotein, which consequently obstruct the viral replication, and in our interaction analysis, all of the four peptides can form hydrogen bonds with Cys145. Moreover, compared to MR-1, MR-2 and MR-3 formed additional hydrogen bonds with T21, S46, P168, T169, and G170, respectively and MR-4 only established an extra hydrogen bond with S46. Figures 3B,D,F,H showed the binding surface of MRs 1-4 and Mpro, from which we observed that the four screened peptides can match well with the binding pocket of Mpro. Figure 4 displayed the binding modes of RBD with MRs 1–4. As shown in Figures 4A,C,E,G, the α-helix region of four peptides was the main region to interact with RBD. All of them formed stabilizing hydrogen bonds with crucial residues K417, Y449, and T500 in the active site of RBD. Moreover, MR-1 formed a hydrogen bond with N487 and D420, respectively. MR-2 and MR-3 only formed a hydrogen bond with N487, while MR-4 formed a hydrogen bond with D420. In addition, it can also be observed from Figures 4B,D,F,H that MRs 1-4 can be perfectly accommodated within the active pocket of RBD. Overall, these docking results indicate that MRs 1-4 are able to simultaneously interact with key residues of Mpro and RBD thereby leading to the possible inhibition of the target proteins.

### MD simulations

**FIGURE 5:**
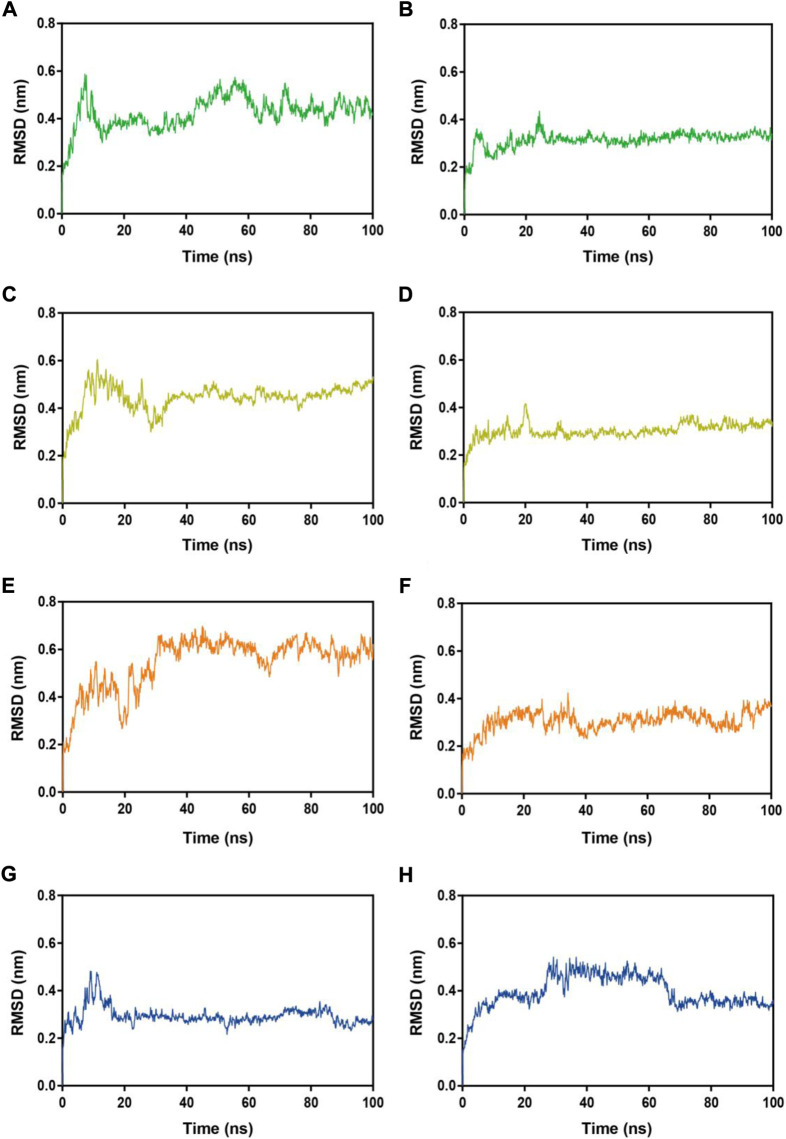
RMSDs of Cα atoms during MD-simulation. **(A**,**B)** MR-1 in complex with Mpro and RBD, respectively; **(C**,**D)** MR-2 in complex with Mpro and RBD, respectively; **(E**,**F)** MR-3 in complex with Mpro and RBD, respectively; **(G**,**H)** MR-4 in complex with Mpro and RBD, respectively.

**FIGURE 6:**
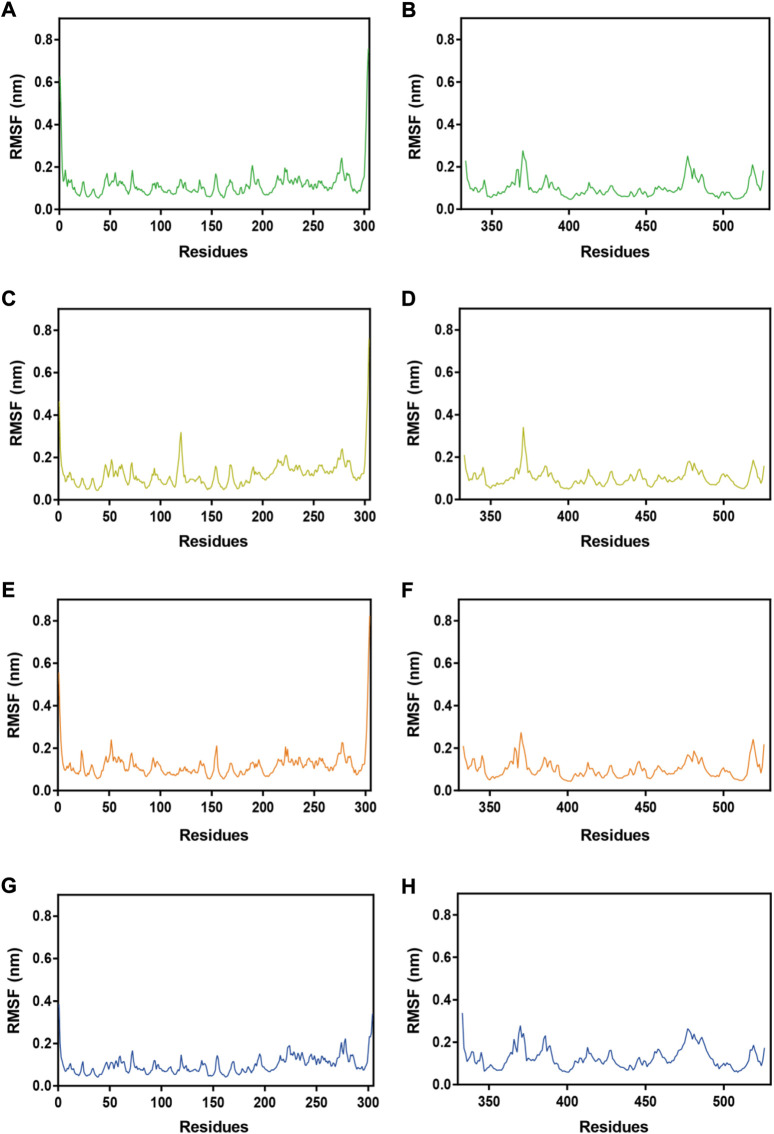
RMSFs of Cα atoms during MD-simulation. **(A**,**B)** MR-1 in complex with Mpro and RBD, respectively; **(C**,**D)** MR-2 in complex with Mpro and RBD, respectively; **(E**,**F)** MR-3 in complex with Mpro and RBD, respectively; **(G**,**H)** MR-4 in complex with Mpro and RBD, respectively.

Subsequently, MRs 1-4-Mpro complexes and MRs 1-4-RBD complexes were subjected to 100 ns MD simulation studies to explore the stability of systems evolved with time. RMSD serves as a vital factor to describe the stability of a system undergoing MD simulation. Figure 5 depicted RMSDs of the protein backbone (Cα) atoms of MRs 1-4 in complex of Mpro and RBD, respectively. The RMSDs of MRs 1-4-Mpro complexes were discovered to be relatively stable at about 0.45 nm, 0.48 nm, 0.6 nm, and 0.3 nm, respectively. The RMSDs of MRs 1-4-RBD complexes were discovered to stabilize at about 0.35 nm. Some fluctuations were observed in the beginning of all these eight complex systems, and then the complexes gradually tended to equilibrium. This suggests that MRs 1-4-Mpro and MRs 1-4-RBD complexes are fairly stable through the simulations and MRs 1-4 can simultaneously bind stably with Mpro and RBD throughout the simulations. RMSF of Cα atoms of the protein residues was calculated to obtain residues contact information between target proteins and peptides. A higher value suggests more flexibility of the amino acid and the lower fluctuations means a good stability. As shown in Figure 6, residues in the active site of Mpro and RBD were not found too flexible during simulations, which fluctuated with intensity less than 0.35 nm. Both RMSD and RMSF stabilities are essential to reflect good binding affinities and stability. Based on the above analysis, we can conclude that MRs 1-4 can stably bind to the active pocket of the Mpro and RBD. Thus, MRs 1-4 might be effective dual RBD/Mpro-targeting inhibitors.

### Identification of dual-targeting peptides

**FIGURE 7:**
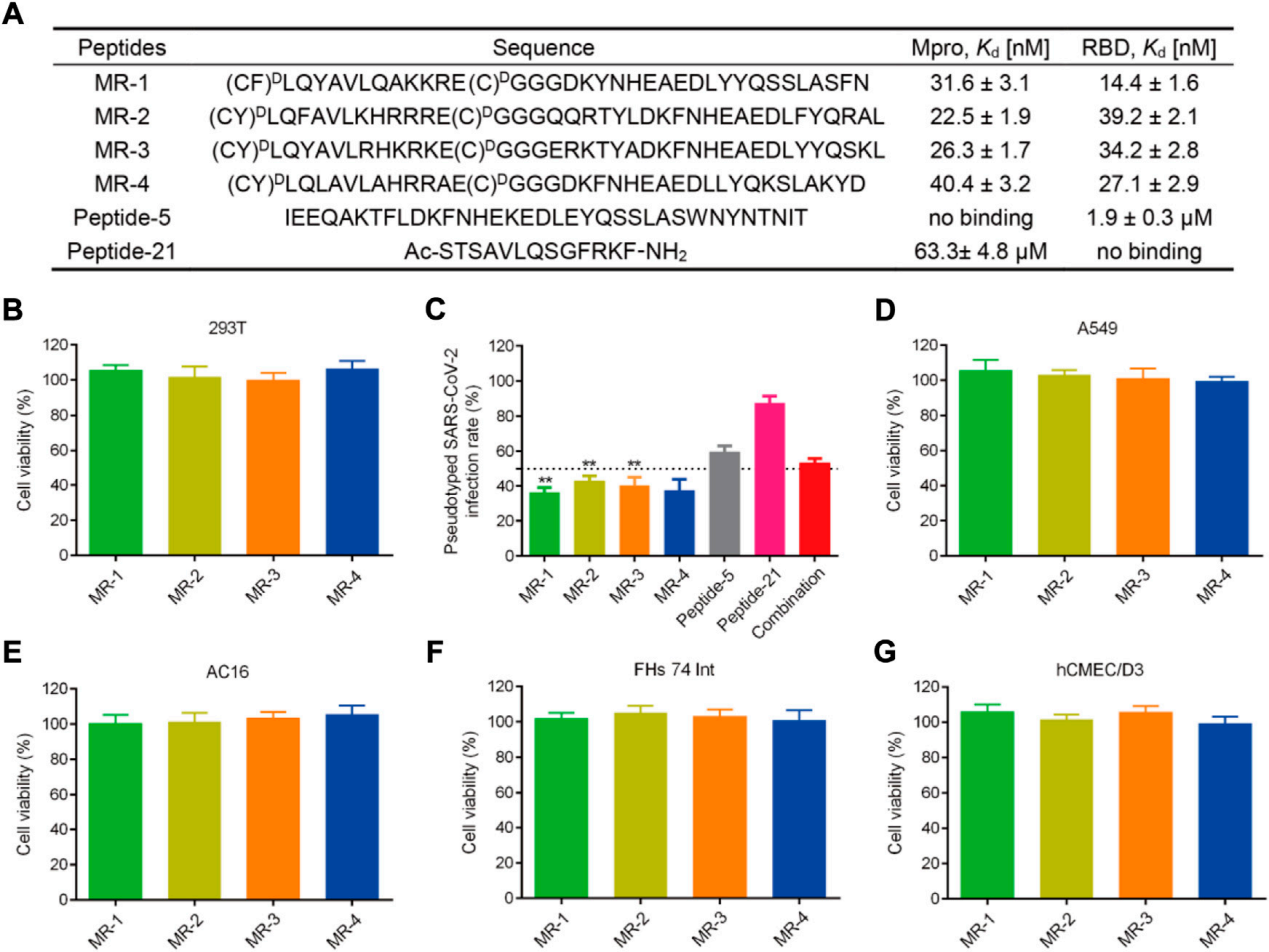
**(A)** Sequences of the peptides and binding affinities of peptides to the Mpro and RBD proteins. MST data shown represent the mean ± SD (*n* = 3). Peptide-5 and Peptide-21 served as the positive controls. **(B)** Effects of MRs 1-4 on the viability of 293T cells. Cells were treated with peptides at a concentration of 50 μM for 48 h, MTT assay was used to detect the cytotoxicity of peptides to cells. The results are represented as mean ± SD (*n* = 3). **(C)** Infection rate of the pseudotyped SARS-CoV-2 virus. MRs 1-4, Peptide-5, Peptide-21, and the combination of Peptide-5 and Peptide-21 were tested at a concentration of 5 μM, respectively. **p < 0.01 means a significant difference *versus* Peptide-21. **(D**–**G)** Effects of MRs 1-4 on the viability of A549, AC16, FHs 74 Int and hCMEC/D3 cells. Cells were treated with 50 μM of MRs 1-4 for 48 h, MTT assay was used to detect the cytotoxicity of peptides to cells. The results are represented as mean ± SD (*n* = 3).

We then determined the binding abilities of MRs 1–4 to the Mpro and RBD proteins using the MST assay. Figure 7A showed that MRs 1–4 displayed strong binding affinities to both Mpro and RBD proteins with *K*_d_ values in a nanomolar range of 22.5–40.4 nM and 14.4–39.2 nM, respectively. Furthermore, we detected the binding affinity of peptide-21 (a positive Mpro targeting inhibitor) (Ullrich et al., 2021) and peptide-5 (a positive RBD targeting inhibitor) (Maas et al., 2021) to Mpro and RBD proteins, respectively. We found that peptide-21 exhibited strong binding affinity to Mpro protein (*K*_d_ = 63.3 ± 4.8 μM) and no binding affinity to RBD protein, while peptide-5 showed strong binding affinity to RBD protein (*K*_d_ = 1.9 ± 0.3 µM) and no binding affinity to Mpro protein. By comparison, the binding of MRs 1-4 to Mpro and RBD proteins was increased remarkably than that of peptide-21 and peptide-5 by about 1,566- to 2,813- and 48- to 131-fold**,** respectively.

### Inhibitory effects of MRs 1-4 on mpro

To further evaluate the inhibitory effects of MRs 1-4 on Mpro, a FRET-based Mpro enzymatic inhibition assay was performed. Ebselen was selected as a positive control drug (Jin et al., 2020). As shown in Supplementary Table S2, MRs 1-4 and Ebselen inhibited the Mpro activities with IC_50_ values of 28.2 ± 2.5, 19.4 ± 1.6, 22.1 ± 1.8, 35.6 ± 2.7, and 661.2 ± 53.5 nM, respectively. It is worth mentioning that MRs 1-4 exhibited much stronger inhibitory effects on Mpro than that of Ebselen.

### Blockage of pseudovirus infection

SARS-CoV-2 is a highly pathogenic virus that requires to conduct research on it in biosafety level 3 (BSL3) or BSL4 laboratories. A pseudovirus provides an alternative choice to the SARS-CoV-2 virus, which can be used at BSL2 and is ideal for screening of therapeutic agents targeting viral entry (Millet et al., 2019; Larue et al., 2021). Therefore, we examined the antiviral potencies of MRs 1-4 against the SARS-CoV-2 virus by conducting the SARS-CoV-2 pseudovirus infection assay. Firstly, the cytotoxicity of MRs 1-4 was examined. Figure 7B revealed that MRs 1-4 were nontoxic to 293T cells at a concentration of 50 µM. The pseudovirus infection assay was then performed at a concentration of 5 μM, which was a nontoxic concentration. As shown in Figure 7C, MRs 1-4 displayed >50% inhibition against the pseudotyped SARS-CoV-2 virus. However, peptide-5 showed about 40% inhibition of SARS-CoV-2 infection while peptide-21 inhibited viral infection by <20%. In addition, we found that a combination of peptide-5 and peptide-21 resulted in slightly less than 50% inhibition against the pseudotyped SARS-CoV-2 virus. MRs 1-4 were more effective in suppressing viral infection, compared with peptide-5, peptide-21 and the combination of them. Further experiments showed that MRs 1-4 can inhibit pseudotyped SARS-CoV-2 infection in a concentration-dependent manner (Supplementary Figure S1). These results suggest that MRs 1-4, a class of dual RBD/Mpro-targeting agents, hold potential and effective therapeutic effects in the treatment of SARS-CoV-2 infection.

### Safety profiles of MRs 1-4

Respiratory disease is the hallmark of COVID-19, meanwhile gastrointestinal symptoms, cardiac injury, and neurologic symptoms may occur. SARS-CoV-2 was found in relevant organs including lungs, intestines, heart, and brain (Akhmerov and Marbán, 2020; Yang W. et al., 2020; Mao et al., 2020; Graham et al., 2022). Therefore, to further investigate the safety of MRs 1-4, a MTT assay was conducted to detect the cytotoxicity of MRs 1-4 to A549, FHs 74 Int, AC16, and hCME/CD3 which are in correspondence to the symptoms mentioned above. As shown in Figures 7D–G, after being treated with MRs 1-4 for 48 h with a concentration of 50 μM, it displayed negligible effect on the viability of A549, FHs 74 Int, AC16 and hCMEC/D3 cells, suggesting the excellent safety of MRs 1-4.

## Discussion

RBD and Mpro are closely associated with the cellular entry and viral replication of SARS-CoV-2, and the inhibitors simultaneously targeting RBD and Mpro will effectively inhibit SARS-CoV-2 infection. However, up to date, few dual RBD/Mpro-targeting inhibitors have been reported. In this study, four novel and potent dual RBD/Mpro-targeting peptides, MRs 1-4, were discovered by a multiple virtual screening strategy combining molecular docking-based screening and MD simulation. Interaction analysis demonstrated that MRs 1-4 can block the whole RBD binding surface by interacting with crucial residues K417, D420, N487, Y449, and T500, and form critical hydrogen bonding interactions with C145 at the catalytic domain of Mpro. MD simulations indicated that MRs 1-4 can simultaneously bind stably with RBD and Mpro with no much deviation in the key amino acids of active sites of them, Therefore, the four screened peptides may be effective dual RBD/Mpro inhibitors. Further MST assay showed that MRs 1-4 exhibited strong *in vitro* affinities to both RBD and Mpro in the nanomolar range of 14.4–39.2 nM and 22.5–40.4 nM, respectively, which was enhanced remarkably than that of peptide-21 and peptide-5 by about 1,566- to 2,813- and 48- to 131-fold**,** respectively. Pseudotyped virus infection assay showed that MRs 1-4 displayed >50% inhibition against pseudotyped SARS-CoV-2 virus without **detectable cellular toxicity**. **In addition, it showed** negligible cytotoxicity to A549, FHs 74 Int, AC16 and hCMEC/D3 cells**, suggesting their safety in the biological system.** In summary, these results suggest that MRs 1-4 is a class of potent dual RBD/Mpro-targeting, low-toxic and high-efficacy agents that could be potential to prevent and treat the infection of SARS-CoV-2.

Cyclic peptides are an important class of drugs, which have unique biochemical and therapeutic properties for pharmaceutical applications (Abdalla and McGaw, 2018). In the past two decades, cyclic peptide-based drugs have increasingly been developed, which confirms the common perception that cyclic peptides have ease of manufacture as small molecules, high binding affinities and low metabolic toxicity as antibodies (Zhang and Chen, 2022). Compared with linear peptides, cyclic peptides showed strong enhancement in receptor binding affinity, specificity, and stability, partly due to the reduction of their conformational freedom (Katsara et al., 2006; Sohrabi et al., 2020). However, the lability of the disulfide bond to reducing agents in plasma can reduce the biological activity of disulfide-containing cyclic peptides and limit their usefulness as therapeutics (Muttenthaler et al., 2010a; Olson et al., 2016). In our follow-up study, to address this concern, substitution of the disulfide bond in MRs 1-4 with a variety of different moieties will be explored, including the use of lactam, (Hargittai et al., 2000) dicarba, (MacRaild et al., 2009) diselenide, (Muttenthaler et al., 2010b) and thioether bridges (Schramme et al., 2008) to enhance the plasma stability. Moreover, since MRs 1-4 can inhibit the activity of Mpro after their entry into host cells, side-chain modification (introducing non-natural hydrophobic groups, *etc.*) or N-methylation of the peptide backbone will be conducted to improve the membrane permeability of MRs 1-4 and thereby improve their activity. (Buckton et al., 2021). Peptides with better pharmacokinetic properties and improved activity after structural optimization could be suitable for nasal gel formulation and could directly distribute to the respiratory system through dry powder or atomization (Lewis and Richard, 2015). We believe that this class of dual RBD/Mpro-targeting peptides may be valuable in treatment of COVID-19 disease.

## Data Availability

The original contributions presented in the study are included in the article/Supplementary Material, further inquiries can be directed to the corresponding authors.
