# Supplementary material for: Dual-targeting cyclic peptides of receptor-binding domain (RBD) and main protease (Mpro) as potential drug leads for the treatment of SARS-CoV-2 infection
Source: Front Pharmacol. 2022 Oct 19;13:1041331. doi: 10.3389/fphar.2022.1041331 (PMC9627161; doi:10.3389/fphar.2022.1041331)
Supplement: Supplementary file 1 [file DataSheet1.docx]

Supplementary Material

**Table S1. The docking scores of the screened peptides against Mpro and RBD**

|  | Mpro | RBD |
| --- | --- | --- |
| Name | Binding free energy*^a^* | Binding free energy |
|  | (kcal/mol) | (kcal/mol) |
| MR-1 | -13.76 | -13.85 |
| MR-2 | -13.92 | -13.73 |
| MR-3 | -13.81 | -13.79 |
| MR-4 | -13.71 | -13.81 |

*^a^*Binding free energy between the peptide and the target (lower binding free energies indicate stronger binding affinities).

**Table S2. Inhibitory effects of MRs 1-4 and Ebselen on Mpro**

|  | MR-1 | MR-2 | MR-3 | MR-4 | Ebselen |
| --- | --- | --- | --- | --- | --- |
| IC_50_ (nM) ± SD*^a^* | 28.2 ± 2.5 | 19.4 ± 1.6 | 22.1 ± 1.8 | 35.6 ± 2.7 | 661.2 ± 53.5 |

*^a^*The results are representative of three independent experiments and are expressed as mean ± SD.


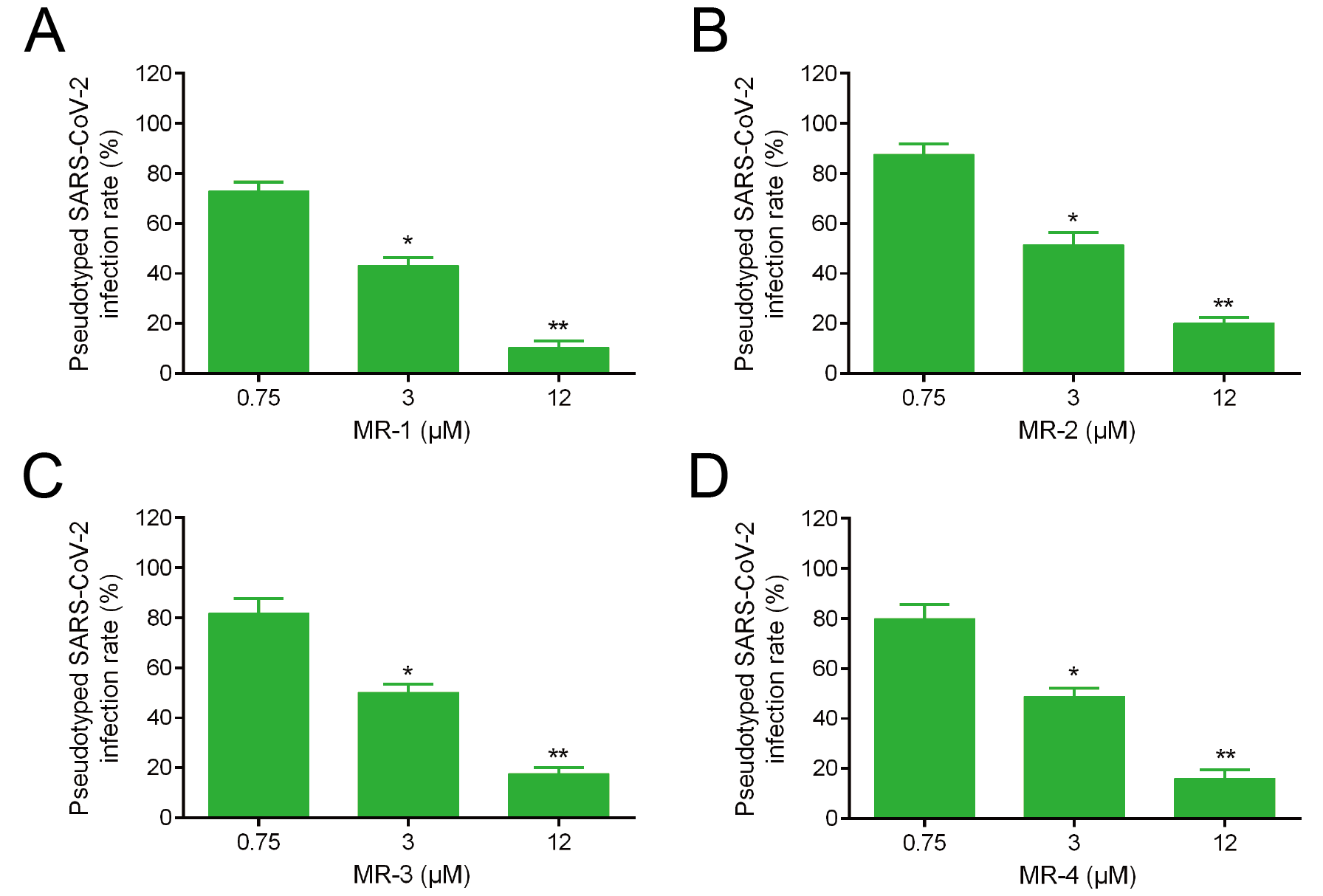


Figure S1. (A-D) Concentration-dependent (0.75-12 μM) assay of MRs 1-4. **p* < 0.05, ***p* < 0.01 versus 0.75 μM. The results are represented as mean ± SD (n = 3).
